# Genomic Characterization of a Reassortant A/H1N8 Avian Influenza Virus in South American Migratory Shorebirds

**DOI:** 10.3390/pathogens15101039

**Published:** 2026-10-01

**Authors:** Gustavo de Oliveira Fenner, Luciano Matsumiya Thomazelli, Erick Gustavo Dorlass, Ana Karolina Antunes Eisen, Nicole Almeida dos Reis, Edison Luiz Durigon Junior, Thais Pailo de Carvalho, Telma Alves Monezi, Desyrée Yumiko Sadoyama Rangel Ozaki, Angie Lorena Castillo Silva, Antônio Coimbra de Brum, Mateus Luís Haas, Júlia Victória Grohmann Finger, Maria Virginia Petry, Stephanie Feigin, Larry Niles, Mario Gabriel Lopes Barboza, Fernanda Panício Vizu, Theo Kraiser, Edison Luiz Durigon, Jansen de Araujo

**Affiliations:** 1 Laboratório de Pesquisas em Vírus Emergentes—LPVE, Departamento de Microbiologia, Instituto de Ciências Biomédicas, Universidade de São Paulo, São Paulo 05508-000, SP, Brazil; gustavo_fenner@icb.usp.br (G.d.O.F.); lucmt@usp.br (L.M.T.); nicole.reis.esib@esib.butantan.gov.br (N.A.d.R.); thaispailocarvalho@gmail.com (T.P.d.C.); telmaamo@usp.br (T.A.M.); desyumi.walker@gmail.com (D.Y.S.R.O.); angiesilva0@gmail.com (A.L.C.S.); fernandap.vizu@icb.usp.br (F.P.V.); theokraiser@gmail.com (T.K.); 2 Laboratório de Virologia Clínica e Molecular—LVCM, Departamento de Microbiologia, Instituto de Ciências Biomédicas, Universidade de São Paulo, São Paulo 05508-000, SP, Brazil; erick.dorlass@einstein.br (E.G.D.); anaeisen@usp.br (A.K.A.E.); eldurigo@outlook.com (E.L.D.J.); mario.lopes98@usp.br (M.G.L.B.); eldurigo@usp.br (E.L.D.); 3 Einstein Hospital Israelita, São Paulo 05652-900, SP, Brazil; 4 Laboratório de Ornitologia e Animais Marinhos, Universidade do Vale do Rio do Sinos (UNISINOS), São Leopoldo 93022-750, RS, Brazil; acbrum@gmail.com (A.C.d.B.); mateushaas1@gmail.com (M.L.H.); victoriafinger@hotmail.com (J.V.G.F.); vpetry@unisinos.br (M.V.P.); 5 Wildlife Restoration Partnerships, 109 Market Lane, Greenwich, NJ 08323, USA; stephaniefeigin@gmail.com (S.F.); larry.niles@gmail.com (L.N.); 6 Institut Pasteur de São Paulo—IPSP, São Paulo 05508-020, SP, Brazil

**Keywords:** avian influenza, South America, migratory birds, genomic surveillance, reassortment

## Abstract

Avian influenza viruses (AIVs) circulate widely among migratory birds, where frequent reassortment drives the emergence of novel genomic constellations. However, the evolutionary pathways underlying these events remain poorly understood in South America due to historically limited genomic surveillance. Here, we report the detection and evolutionary reconstruction of a low pathogenic avian influenza virus (LPAI) subtype H1N8 isolated from a White-rumped Sandpiper (*Calidris fuscicollis*) sampled at Lagoa do Peixe National Park (PNLP), southern Brazil, an important migratory stopover along the Atlantic Flyway. Between 2023 and 2025, 1787 samples were collected from wild birds, resulting in six AIV-positive detections during April 2024, from which one H1N8 isolate was successfully recovered and whole-genome sequenced. Genomic analyses identified a reassortant virus sharing five internal gene segments (*PB2*, *PB1*, *PA*, *NP*, and *MP*) with a previously described H11N2 virus detected at the same site and host species, whereas the *HA*, *NA*, and *NS* segments belonged to distinct South American lineages. Bayesian temporal reconstruction, discrete phylogeographic analyses, ancestral-state reconstruction, and reassortment inference consistently supported the persistence of a shared North American-derived genomic backbone followed by the acquisition of locally circulating South American gene segments. Although based on a single isolate, these findings provide an evolutionary snapshot of a reassortment event in South American migratory birds and reinforce PNLP as a key site for long-term avian influenza virus surveillance.

## 1. Introduction

Avian Influenza Viruses (AIVs) are emerging infectious agents of the genus *Alphainfluenzavirus influenzae* that infect a wide range of avian species, their primary reservoirs [1]. Annually, migratory birds connect ecosystems, transporting a diverse pool of AIV genes and generating genetic exchange at stopover sites [2]. These ecological interactions promote genetic reassortment, where different subtypes co-infecting a single host exchange gene segment to form novel viruses. This process, along with the accumulation of point mutations, are primary drivers of viral evolution [3].

These mechanisms provide a baseline for understanding the evolutionary history of the pandemic diseases such as Spanish flu in 1918 (H1N1), which has been proposed to involve reassortment between an established human H1 lineage and other AIVs, with the acquisition of avian-origin gene segments contributing to the emergence of the pandemic virus [4,5]. Similarly, the 2009 H1N1 pandemic virus resulted from a complex reassortment history involving swine, human, and avian influenza gene pools, with its *PB2* and *PA* segments ultimately derived from avian influenza viruses through previous reassortment events in swine [6]. Although this event did not result from the direct transmission of an AIV into humans, it illustrates how avian influenza viruses can contribute genetic material to the emergence of novel viruses with pandemic potential.

South America has historically been underrepresented in surveillance. Despite the region’s immense biodiversity and conservation sites, it has been marked by temporal and geographical gaps [7,8], with predominantly low pathogenic avian influenza (LPAI) detections. AIV circulation in the continent is mainly sustained through transmission between waterfowl [9]. Genetic exchange between South and North American subtypes, mostly occurring at migratory windows [10,11], defines the continent’s fundamental evolutionary dynamics.

Worldwide, highly pathogenic avian influenza (HPAI) strains have been consistently detected in Eurasian regions, where most of these strains emerged [12]. However, in 2020, H5N1 clade 2.3.4.4b started an expansion, reaching North America in late 2021, and subsequently causing an unprecedented panzootic in South America [13]. The incursion was well documented, showing the virus expansion from the Pacific coast toward the Atlantic side [14,15], where it caused mass mortality events and alarming signs of mammal-to-mammal transmission.

Southern Brazil along the Atlantic coastal region was also affected. Brazil reported its first case in May 2023, with detection in a Cabot’s tern specimen in Marataízes, Espírito Santo. Most detections occurred in seabirds throughout the southern region, with records in backyard poultry, other wild birds, and marine mammals in late 2023 [16,17].

The Lagoa do Peixe National Park (PNLP), located on the Atlantic coast of Rio Grande do Sul, southern Brazil, is one of the main sites for migratory shorebirds of Brazil [18]. These birds maintain a migratory connection with Delaware Bay (DE) in the United States [19], a globally recognized hotspot for surveillance [20]. This ecological link positions PNLP as a critical site for surveillance of avian pathogens [21]. This raised the urgent need to determine whether HPAI or other AIVs were being disseminated through PNLP.

In January 2023, active surveillance at PNLP resulted in the detection of a low pathogenic A/H11N2 virus in a White-rumped Sandpiper (*Calidris fuscicollis*). Genomic analyses demonstrated that H11N2 possessed a predominantly North American genomic constellation, with all internal gene segments clustering with viruses circulating in migratory shorebirds sampled at Delaware Bay between 2020 and 2022, particularly *Calidris* spp. Notably, the PB1 segment was closely related to H5N1 Am 1.2 genotype viruses, highlighting the circulation of this lineage in migratory birds in Brazil [22].

Following this detection, the Laboratory for Research on Emerging Viruses (LPVE) maintained continuous surveillance at PNLP between 2023 and 2025. Although no highly pathogenic avian influenza viruses were detected, these surveillance efforts led to the identification of a reassortant H1N8 virus in the same host species. Additionally, genomic characterization revealed that most of the internal gene segments were highly similar to those of the prior H11N2 virus, revealing a strong connection between these two viruses and providing an opportunity to investigate their evolutionary relationship in a discrete snapshot of reassortment.

## 2. Materials and Methods

### 2.1. Sample Collection and Processing

Surveillance and sample collection were conducted during four expeditions between December 2023 and April 2025 at PNLP (31°25′ S, 51°08′ W). The sampling strategy included the collection of oropharyngeal and cloacal swabs and fresh fecal samples from the environment. For each captured species, oropharyngeal and cloacal swabs were collected from the same bird and combined in a single cryotube. Additional precautions were adopted for environmental fecal sampling. Whenever possible, fresh fecal samples were collected from areas occupied by monospecific shorebird flocks. Flocks were observed before and during sampling, and recently deposited feces were collected after birds moved from their resting or foraging locations. All procedures were performed using appropriate personal protective equipment (PPE) by all researchers involved.

Each sample was placed in a cryotube containing Viral Transport Medium (VTM), consisting of PBS supplemented with antimicrobial agents (200 μg/mL penicillin G, 200 μg/mL streptomycin, 25 μg/mL fungizone, and 6 μg/mL gentamicin) and 10% glycerol. Following collection, samples were immediately stored in a liquid nitrogen dry shipper and transported to the LPVE at the University of São Paulo.

Samples were initially centrifuged, and the supernatant was used for RNA extraction with a MagMax™-96 RNA Isolation Kit (Ambion, Austin, TX, USA). Following extraction, all samples were screened for a conserved region of the matrix gene [10] using the AgPath-ID™ One-Step RT-PCR Kit (Applied Biosystems, Foster City, CA, USA). Samples that tested positive for the matrix (MP) gene were selected for viral propagation. Virus isolation was performed using standard methods in 10-day-old embryonated chicken eggs [23]. Three days’ post-inoculation, chicken embryos were observed for mortality signs prior to collection, and allantoic fluid was harvested and re-tested by RT-qPCR to confirm viral isolation. These procedures were performed at biosafety laboratory level 3 (BSL-3^+^) from the Parasitology department from Biomedical Sciences Institute II at University of São Paulo.

### 2.2. Amplification, Next-Generation Sequencing, Genome Assembly, and Identification

All extracted RNAs were submitted to Sanger sequencing for an initial genomic characterization. Samples had HA and MP partial genes amplified using primers described by Hoffmann (2001) and sequenced using standard Sanger methods [24,25]. All eight segments were amplified using the universal primers described by Zhou et al., which target conserved regions at the 3′ and 5′ termini of all IAVs gene segments [26]. The RT-PCR was carried out using the SuperScript™ III One-Step RT-PCR System with Platinum™ Taq DNA Polymerase (Invitrogen™, Waltham, MA, USA). Subsequently, libraries were prepared using the Ion Plus Fragment Library Kit (ThermoFisher Scientific, Carlsbad, CA, USA), the chips were loaded with Ion Chef System and sequenced on Ion GeneStudio S5 platform, as previously validated [25]. Raw sequencing data were subjected to quality control and DE NOVO assembly in Geneious Prime^®^ (Version 2024.0) [27], with read trimming and quality filtering performed using the BBDuk2 plugin at a Phred quality threshold of Q20, the highest recommended for Ion Torrent data. The resulting consensus contigs for each gene segment were queried against GenBank using BLASTn^®^ to identify their closest reference sequences. These closest hits were then used as references for a reference-guided mapping of the reads for each segment (accession numbers OQ533061.1 for *PB2*, *PB1*, *PA*, *NP*, and *MP*; OP888904.1 for HA; PP502599.1 for *NA*; and PP829714.1 for *NS*). The final consensus for each segment was manually inspected for quality and accuracy in Geneious Prime, and genome identification was confirmed through BLASTn searches against the NCBI and GISAID databases. The FASTQ files are deposited at https://github.com/gustavofn13/PNLP_H1N8_Pathogens.git (accessed on 1 September 2026).

We defined the “shared genomic backbone” as the set of five internal gene segments (*PB2*, *PB1*, *PA*, *NP*, and *MP*) that were highly similar between the PNLP viruses (≥98% nucleotide identity) and consistently recovered within North American-derived lineages across all segment phylogenies, with bootstrap support >95 and posterior probability >0.98, sharing a recent common ancestor. This backbone was shared among two Delaware strains (2022) and both PNLP viruses. Notably, the PB1 segment was an exception within this, it was carried exclusively by the two PNLP viruses and was not present in the North American viruses that shared the other four internal segments (*PB2*, *PA*, *NP*, and *MP*).

### 2.3. Phylogenetic Analysis and Reassortment Inference

To characterize the evolutionary relationships of the PNLP H1N8 virus, independent phylogenetic datasets were assembled for each of the eight influenza A virus gene segments. The dataset for Maximum Likelihood analysis (ML) contains the 500 closest hits from GISAID, which were used for phylogenetic reconstruction of all gene segments. Complete coding sequences (CDS) from each segment was aligned using the MAFFT algorithm, and phylogenetic trees were inferred using the ML method implemented in IQ-TREE2 (v2.4.0) under the model finder algorithm, and branch support was assessed with 1000 ultra-fast bootstrap (BB) replicates [28].

To formally test for reassortment and identify specific gene acquisitions, we employed TreeSort v0.3.1 [29] (https://github.com/flu-crew/TreeSort (accessed on 1 July 2026), using the mincut method implemented, with equal substitution rates assumed across genomic segments. This method compares the expected and observed genetic divergence between the reference and query segments under the null hypothesis of no reassortment. When this hypothesis was rejected, reassortment events were inferred and mapped onto the branches of the reference phylogeny. A refined dataset comprising approximately 30 informed strains was assembled by selecting the PNLP viruses together with their closest phylogenetic relatives identified in the segment-specific ML analysis. Additional representative genomes were included to preserve the evolutionary context of the focal lineage, while minimizing redundant sampling. TreeSort generated annotated phylogenetic trees in which branches inferred to contain reassortment events were labeled according to the genomic segments involved. These output trees were examined to identify the phylogenetic position of the H1N8 internal gene segments. Reassortment events were inferred by iteratively assigning each of the five shared internal gene segments (*PB2*, *PB1*, *PA*, *NP*, and *MP*) as the reference phylogeny, allowing TreeSort to statistically detect reassortment along the evolutionary branches. Annotated trees and alignments files used for this analysis are provided in Supplementary File S5 and GitHub repository.

### 2.4. Bayesian Phylogenetic Approach

To investigate the evolutionary history of the PNLP H1N8 reassortant virus, we performed Bayesian phylogenetic and phylodynamic analyses. Given the limited genomic surveillance of avian influenza viruses in South America, we integrated our newly obtained genome with all available South American H1 sequences to maximize temporal and phylogeographic resolution. The analyses were conducted in two complementary frameworks. First, the HA gene was used to reconstruct the temporal, geographic, and host-associated history of the South American H1 lineage. Second, independent Bayesian analyses of the five internal gene segments (*PB2*, *PB1*, *PA*, *NP*, and *MP*) shared with the closely related PNLP H11N2 virus [22]. Together, these complementary analyses were used to reconstruct the most parsimonious evolutionary scenario supported by the available data.

#### 2.4.1. Bayesian Reconstruction of the South American H1 Lineage

To contextualize the South American H1 lineage containing the PNLP H1N8 virus, a time-scaled HA dataset was assembled comprising all available South American H1 viruses (*n* = 21; Argentina *n* = 10, Chile *n* = 10, and Brazil *n* = 1 [this study]). To provide proper ancestral evolutionary context, 30 closely related North American H1 sequences identified in the same cluster as South American sequences were included, providing a broader phylogenetic background.

To infer the geographic and host-associated evolutionary history of this lineage, we performed discrete trait analyses using a continuous-time Markov chain (CTMC) model [30,31]. Geographic traits were defined at the country level (Argentina, Brazil, and Chile), and avian host categories were assigned based on common species names. To identify the most parsimonious description of the diffusion process, we applied an asymmetric Bayesian Stochastic Search Variable Selection (BSSVS) to infer state transition rates [32,33,34]. Statistical support for individual transitions was evaluated using Bayes factors (BF), where BF > 100 indicates decisive support, BF > 10 strong support, and BF 3–10 positive support [35]. Complementarily, expected numbers of geographic and host-state transitions were estimated as “Markov jumps” by sampling complete state-transition histories across the posterior distribution of trees [32,36], summarizing the most probable sequence of ancestral changes while integrating phylogenetic uncertainty.

To evaluate the robustness of these inferences to model specification, the discrete-trait analyses were repeated under all four combinations of demographic prior (GMRF Bayesian Skyride and constant-size coalescent) and molecular clock (uncorrelated lognormal relaxed and strict), and the BF and Markov-jump support for each transition was compared across the four model cells. To further assess sensitivity to sampling imbalance, a jackknife analysis was performed by generating five replicate datasets, each randomly removing 25% of the Chilean and Argentinian sequences while holding the Brazilian and North American context sequences constant, so that the number of discrete states remained identical across replicates [37,38,39]. Support for each transition was then compared between the full dataset and the replicates. The same asymmetric BSSVS framework was applied independently to the NA (N8) and NS genes to evaluate geographic transitions for these locally acquired segments.

#### 2.4.2. Bayesian Analyses of Shared Internal Gene Segments

To investigate the evolutionary relationships of the internal gene segments shared by PNLP H1N8 and H11N2 viruses, independent Bayesian analyses were performed for the five internal gene segments (*PB2*, *PB1*, *PA*, *NP*, and *MP*). For each segment, a representative time-scaled dataset of approximately 50 sequences was assembled, comprising the closest phylogenetic relatives identified from maximum-likelihood analyses, including closely related North American H11N2 viruses from Delaware Bay (2022), providing an appropriate evolutionary context for the involved lineages. Similarly, we also applied the asymmetric BSSVS framework described above to each segment independently. Geographic sampling locations, together with the local “PNLP_H11N2” and “PNLP_H1N8” viruses, were defined as discrete traits. This segment-specific approach provided independent statistical support for estimating transition rates and exploring the spatiotemporal diffusion history of the lineages associated with the emergence of the reassortant virus.

#### 2.4.3. Computational Methods and Convergence Assessment

To estimate divergence times, including the time to the most recent common ancestor (tMRCA), Bayesian analyses were conducted in BEAST X v10.5 [40]. Temporal signals were initially evaluated through root-to-tip regression using TempEst v1.5.3 [41]. Time-scaled phylogenies were reconstructed under an uncorrelated lognormal relaxed molecular and strict clock [42], both using the codon-partitioned SDR06 nucleotide substitution model. Demographic history was modeled under the GMRF Bayesian Skyride coalescent prior to accommodate changes in effective population size over time [43]. Alternative analyses under a constant-size coalescent prior were also performed in both relaxed and strict clock models, both evaluating the robustness of topology and tMRCA estimates.

For all Bayesian analyses, two independent Markov chain Monte Carlo (MCMC) chains were run for 50 million generations, sampling parameters and trees every 5000 states. Independent runs were combined using LogCombiner v10.5 following removal of 10% burn-in. Convergence and effective sample sizes (ESS) were assessed in Tracer v1.7.2; all analyses achieved ESS > 200 for key parameters (clock rate, tMRCA, transition rates), indicating adequate sampling. Maximum clade credibility (MCC) trees were summarized using TreeAnnotator and visualized in IcyTree. Final figures were exported as scalable vector graphics (SVG) and edited in Affinity Designer.

## 3. Results

### 3.1. Sample Characterization

A total of 1787 samples were collected from wild birds at the PNLP across five expeditions (December 2023, April 2024, July 2024, October 2024, and April 2025) (Figure S1; Supplementary File S1, Table S1). Sampling sites spanned the coastal strip of the park, ranging approximately from 30.42° S–31.29° S and 50.30° W–50.95° W across expeditions, specific coordinates for every sample are provided in Supplementary File S1 (Table S1). The samples comprised thirty-two oropharyngeal/cloacal swabs and 1755 fresh fecal samples. Most samples were from healthy migratory birds of the order Charadriiformes and the family Scolopacidae, with species, dates and individual molecular test results for every sample provided in Supplementary File S1 (Table S1). Six AIV-positive samples by real-time PCR were detected in fecal samples from *Calidris fuscicollis*, collected during the April 2024 expedition, with cycle thresholds (Cts) ranging from 35.2–39.1 (Supplementary File S1, Table S2). The high Ct values observed in five of the six MP-positive samples indicated low viral RNA levels, which limited the feasibility of virus isolation and further genomic characterization.

Initially, all samples were submitted to partial MP and HA Sanger sequencing, but only PNLP_222-FZS yielded readable sequences. These partial fragments, obtained directly from the original sample, confirmed the HA as an H1 virus and the MP as derived from the PNLP H11N2 lineage (2023). This early evidence, later confirmed by whole-genome sequencing, indicated that at least two distinct lineages were involved in the original field material, prior to any egg passage.

All six positive samples were inoculated into embryonated chicken eggs and subjected to three serial passages. Of these only one isolate (PNLP_222-FZS) was successfully recovered and yielded sufficient viral load for successful whole-genome sequencing (Supplementary File S2), decreasing Ct values from 38 to 27 in the first passage (Supplementary File S1, Table S2), additionally, no embryo mortality was observed during the entire process post 72 h of infection. This virus was further characterized as an H1N8 isolate (GenBank accession numbers: PX063867–PX063874). Initial genome identification identified a reassortant virus, in which five internal gene segments (*PB2*, *PB1*, *PA*, *NP*, and *MP*) were highly similar (98–99% nucleotide identity) to an A/H11N2 virus (OQ533064.1) detected in a prior surveillance effort in January 2023 [22]. Nucleotide identities to the closest related reference strains of each segment are summarized in Table 1. In addition, all shared segments of the H11N2/H1N8 cluster are supported by high bootstrap (>95) values in ML trees and by the molecular clock analysis.

**Figure 1:**
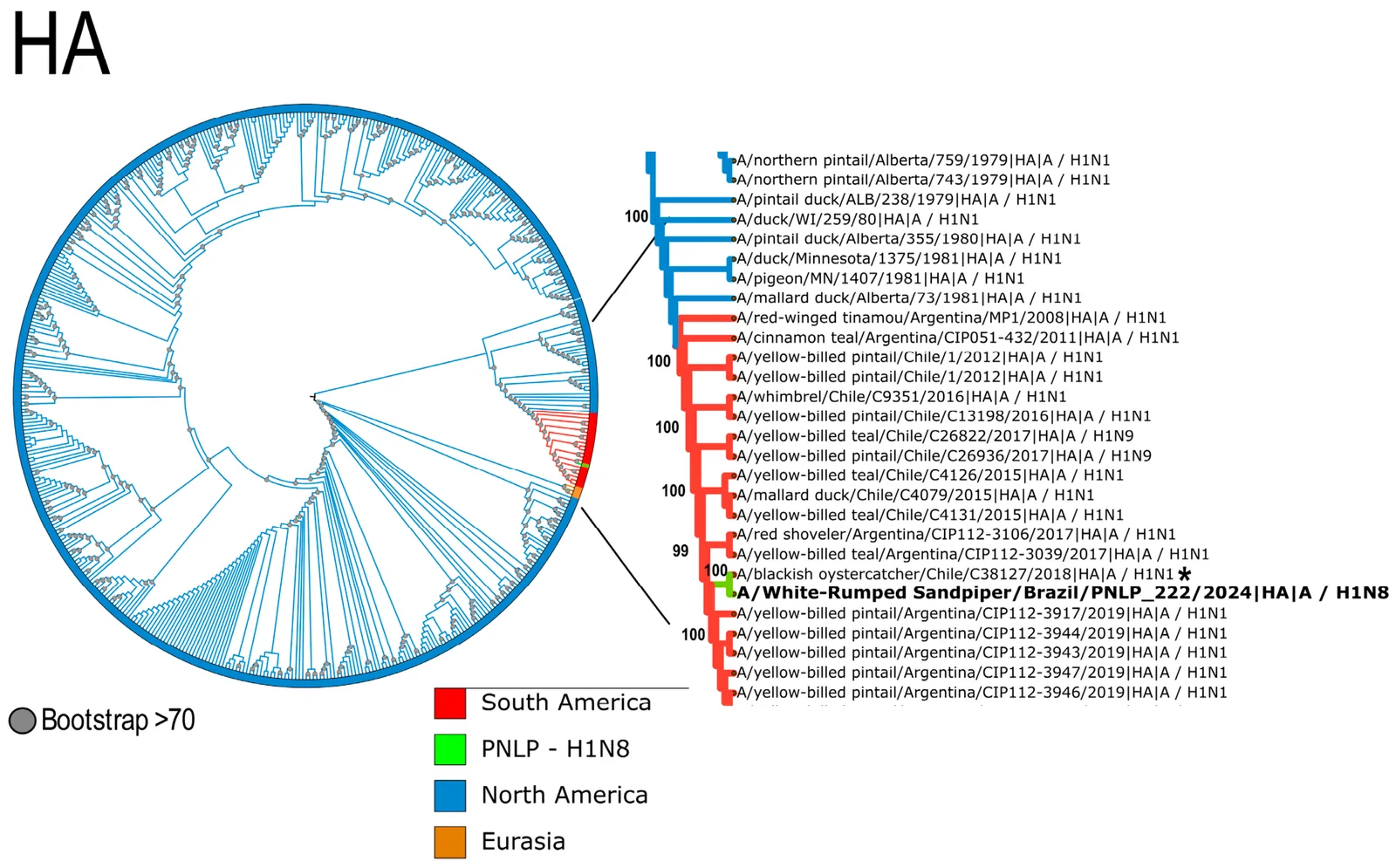
Maximum likelihood phylogenetic tree (500 sequences) of the H1 hemagglutinin (HA) gene segment. Branch colors indicate the geographic origin of the lineages: North America (blue), South America (red), and Eurasia (orange). The H1N8 isolate sequenced in this study (A/White-Rumped Sandpiper/Brazil/PNLP_222/2024) is highlighted in green within the magnified view. Gray circles at the nodes represent bootstrap support values > 70 and numerical bootstraps are shown in magnified view. An asterisk (*) indicates the sequence identified as the corresponding BLASTn hit reported in Table 1.

In contrast, the HA, NA, and NS genes were similar across distinct South American lineages (Table 1). The HA (H1) gene clustered with a small group of viruses detected in Chile and Argentina between 2008 and 2019, and a North American group of older sequences (1970–1981) (Figure 1). The HA of the PNLP H1N8 and other South American H1 viruses possessed the same monobasic cleavage site (PSIQAR/GLF), consistent with a low-pathogenic phenotype.

**Figure 2:**
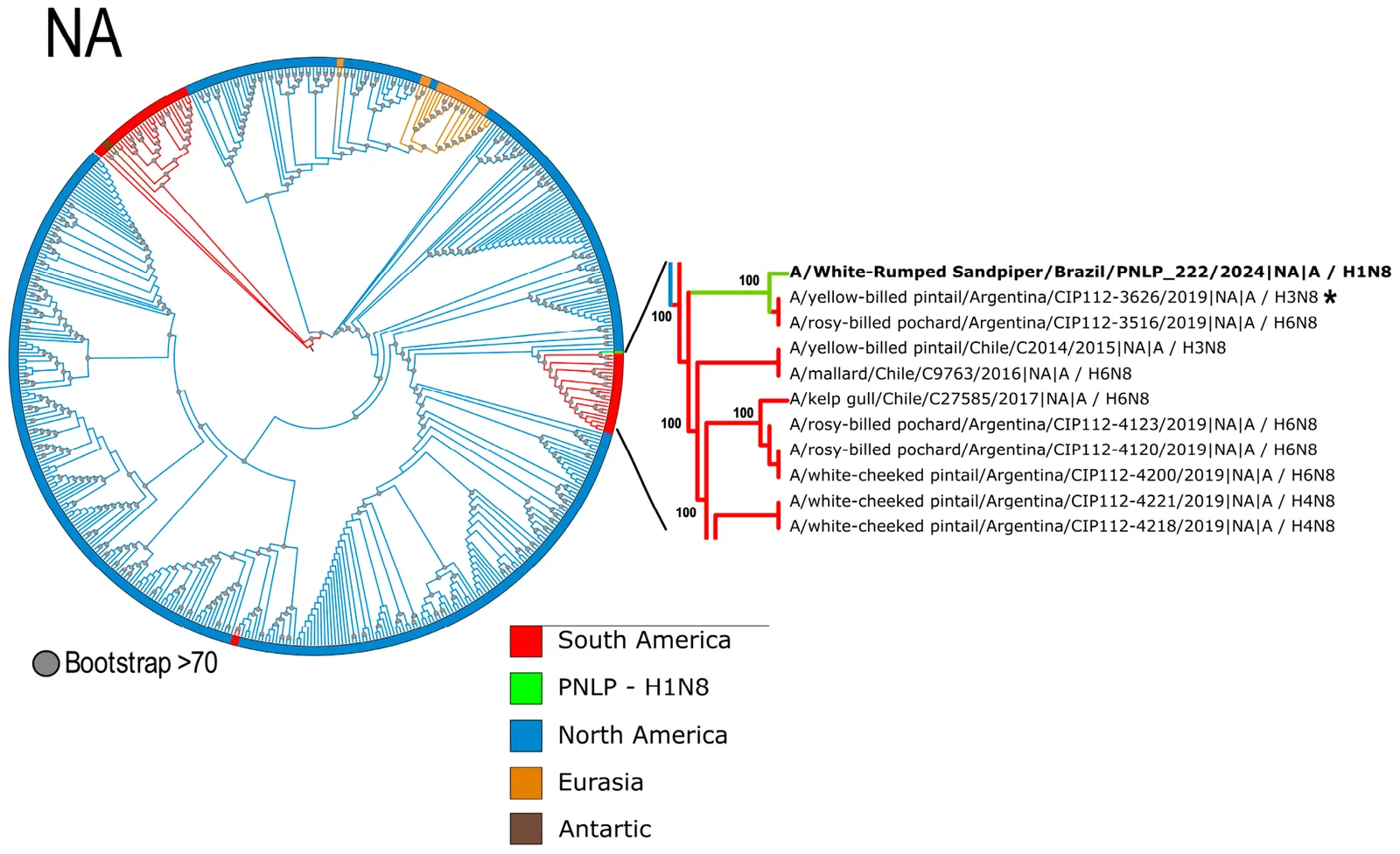
Maximum likelihood phylogenetic tree (500 sequences) of the N8 neuraminidase (NA) gene segment. Branch colors indicate the geographic origin of the lineages: North America (blue), South America (red), and Eurasia (orange). The H1N8 isolate sequenced in this study (A/White-Rumped Sandpiper/Brazil/PNLP_222/2024) is highlighted in green within the magnified view. Gray circles at the nodes represent bootstrap support values > 70 and numerical bootstraps are shown in magnified view. An asterisk (*) indicates the sequence identified as the corresponding BLASTn hit reported in Table 1.

**Figure 3:**
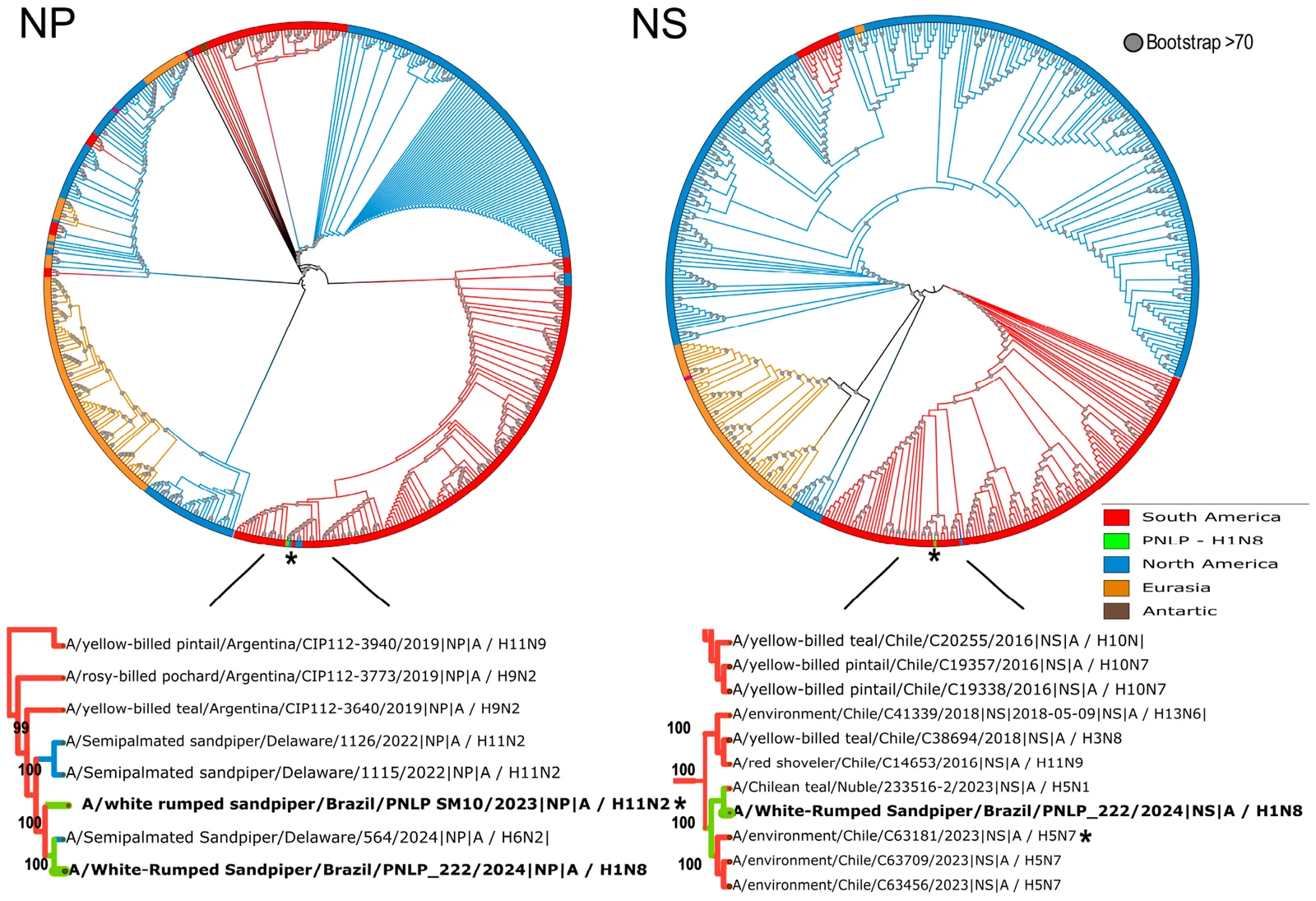
Maximum likelihood phylogenetic trees of the NP and NS gene segments (500 sequences). Branch colors indicate the geographic origin of the lineages: North America (blue), South America (red), and Eurasia (orange). The H1N8 isolate sequenced in this study (A/White-Rumped Sandpiper/Brazil/PNLP_222/2024) is highlighted in green within the magnified views. In the NP tree (left), the H1N8 virus clusters with the previously described PNLP H11N2 virus within a North American sub-clade, supporting a shared internal genomic backbone. In contrast, the NS tree (right) places the H1N8 isolate within a distinct South American lineage, consistent with the reassortment event inferred from the genomic analyses. Gray circles at the nodes represent bootstrap support values > 70 and numerical bootstraps are shown in magnified view. An asterisk (*) indicates the sequence identified as the corresponding BLASTn hit reported in Table 1.

**Figure 4:**
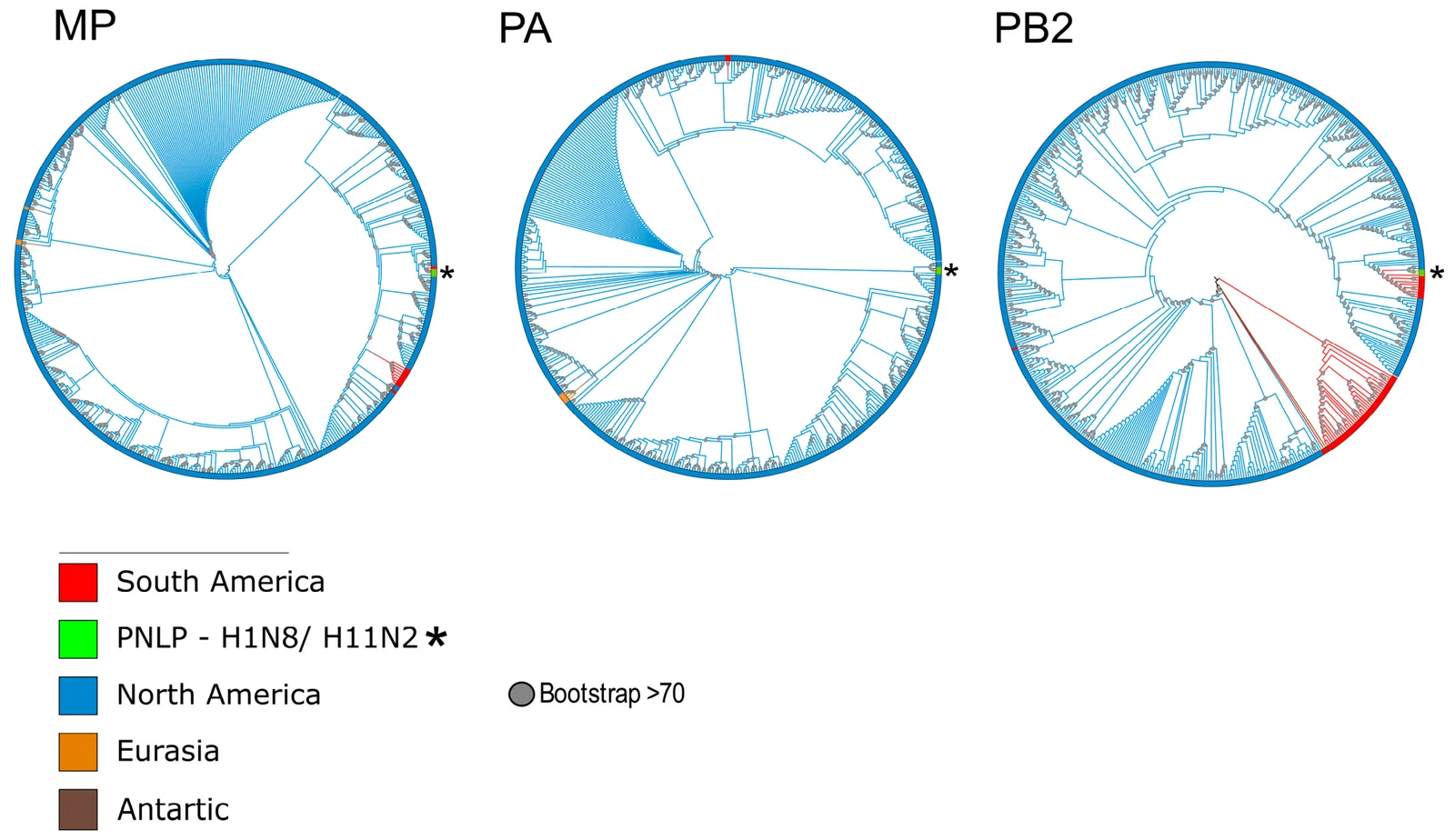
Maximum likelihood phylogenetic trees of the *PB2*, *PA*, and *MP* gene segments (500 sequences for each segment). Branch colors indicate the geographic origin of the lineages: North America (blue), South America (red), Eurasia (orange), and Antarctica (brown). The H1N8 isolate sequenced in this study (A/White-Rumped Sandpiper/Brazil/PNLP_222/2024) and the previously described PNLP H11N2 virus are highlighted in green, illustrating their close phylogenetic relationship within predominantly North American lineages across these internal gene segments. The asterisks (*) indicates the position of the PNLP- H1N8 and PNLP- H11N2 subtypes.

**Figure 5:**
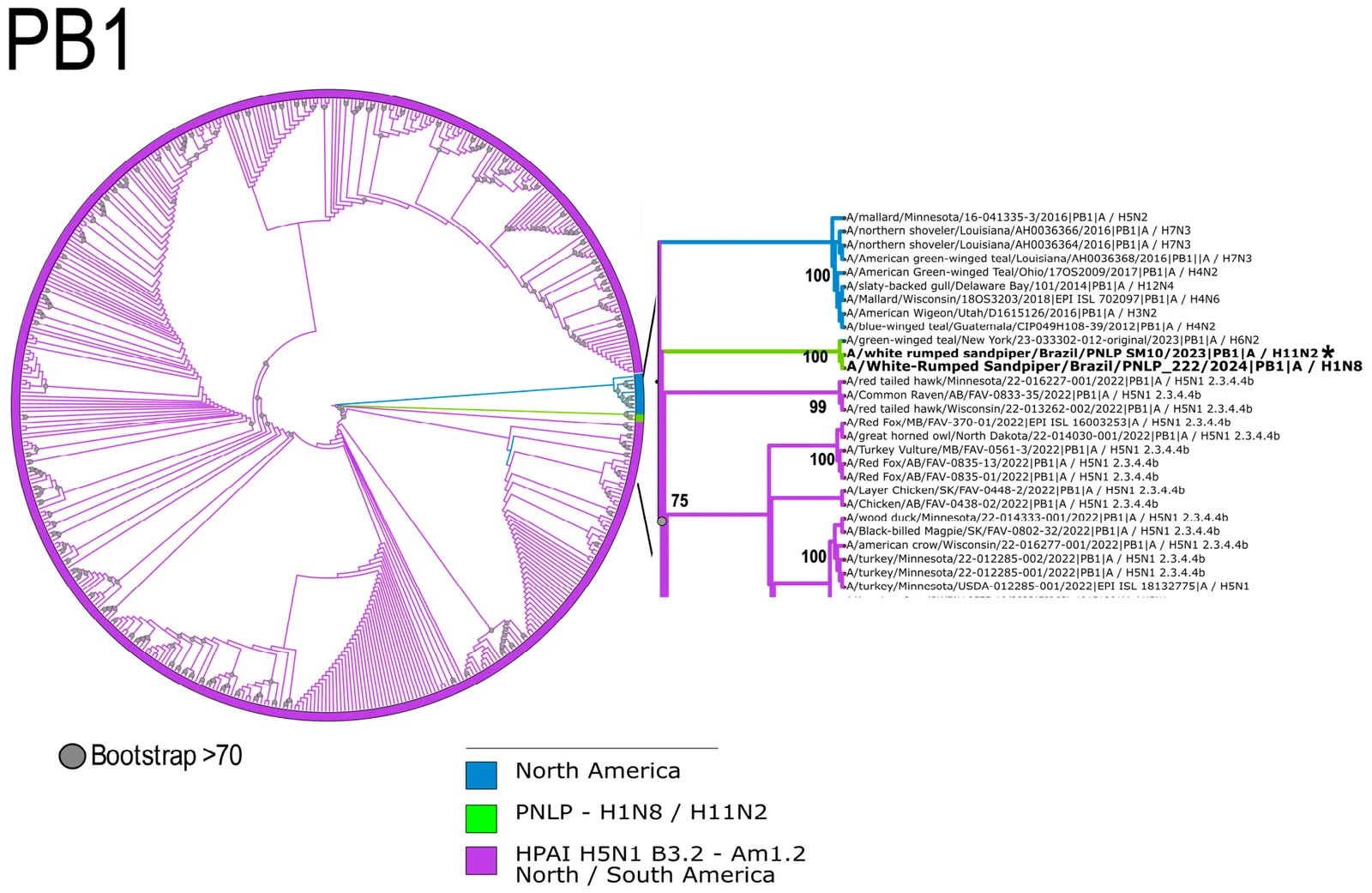
Maximum likelihood phylogenetic tree of the PB1 gene segment (500 sequences). Branch colours indicate North American low pathogenic lineages (blue), HPAI H5N1 clade 2.3.4.4b genotype B3.2 viruses of the Am1.2 lineage (purple), and the viruses sequenced at Lagoa do Peixe National Park (green). The magnified view shows the position of the H1N8 isolate of this study (A/White-Rumped Sandpiper/Brazil/PNLP_222/2024) together with the previously described PNLP H11N2 virus (A/white-rumped sandpiper/Brazil/PNLP_SM10/2023), which branch adjacent to the H5N1 Am1.2 radiation within a broader assemblage of low pathogenic viruses sampled from waterfowl and shorebirds across several North American states. Grey circles at the nodes represent bootstrap support values > 70 and numerical bootstraps are shown in magnified view. An asterisk (*) indicates the sequence identified as the corresponding BLASTn hit reported in Table 1.

The NA (N8) gene tree topology clustered Argentinian viruses sampled in 2019 (Figure 2), although there is another distant local group of N8 sequences that has already been described in other studies [11,44]. The NS segment also belonged to a local clade, clustering with local LPAI H5 viruses detected in environmental samples from Chile in 2023. ML trees of the NP gene placed the H1N8 and H11N2 viruses within a deeply rooted South American clade, which includes subtypes already described at PNLP and other South American countries (Figure 3) [21,45,46]. However, the current NP gene topology is nested within a distinct sub-clade that exhibits recent inter-hemispheric circulation. This group comprises the PNLP viruses and LPAI strains from Delaware (Figure 3). The polymerase genes and matrix segments (*PB2*, *PA* and *MP*) were consistently associated with the North American gene pools (Figure 4), mainly associated with an A/H11N2 from Delaware, 2022. Distinct from this pool, the PB1 gene clustered in a clade associated with HPAI H5N1 sequences from lineage Am1.2, where it was closely related to H5N1 B3.2 sequences from North and South America (Figure 5).

### 3.2. H1 Lineage Evolution in South America

**Figure 6:**
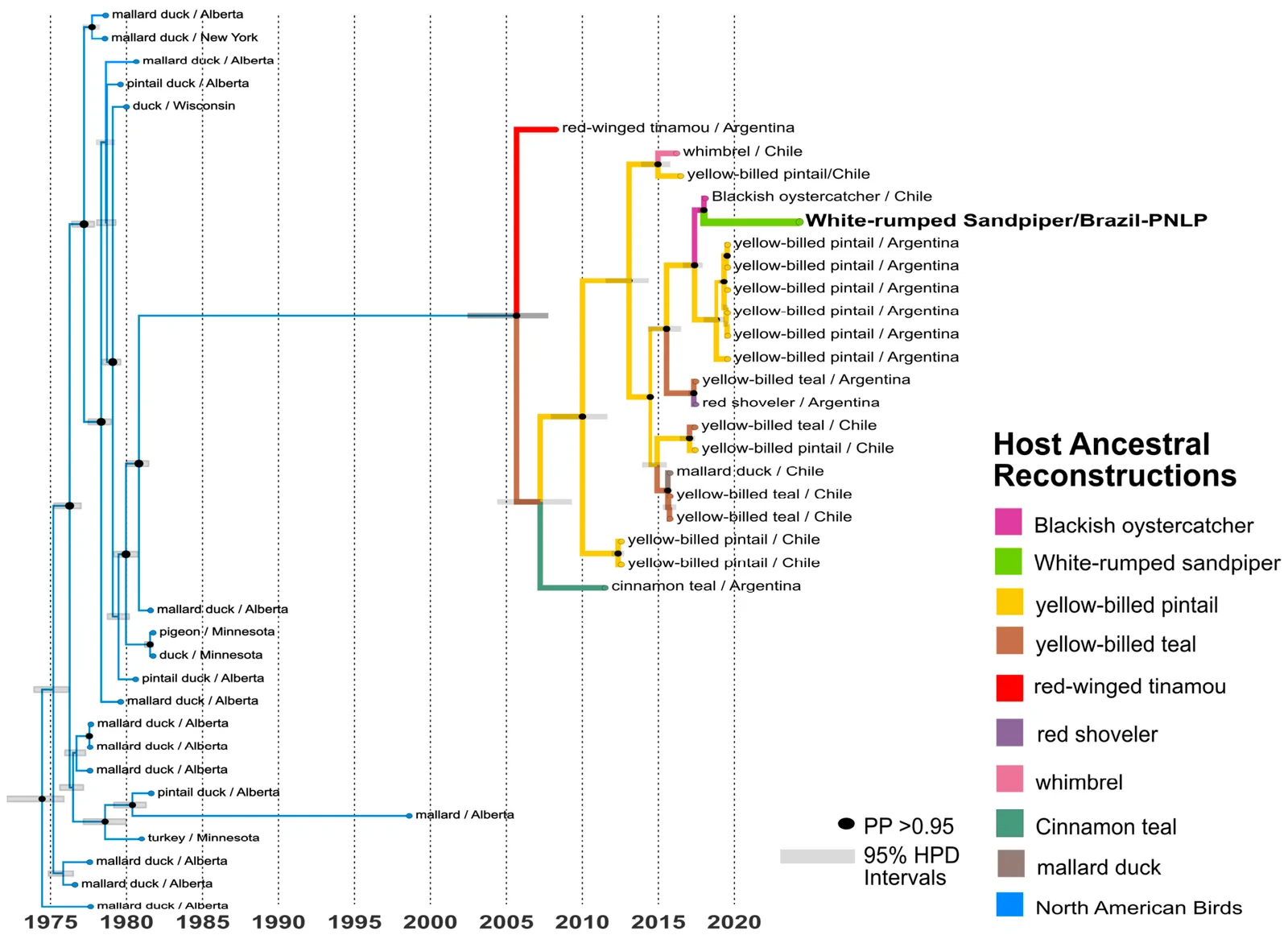
Bayesian ancestral-state reconstruction of the H1 lineage based on host species. Time-scaled maximum clade credibility tree showing ancestral host-state reconstruction. Branch colors indicate the most probable ancestral host species, illustrating the predominance of waterfowl hosts across the lineage, with occasional transitions involving non-waterfowl species, including the Blackish Oystercatcher and the terminal Brazilian isolate detected in a White-rumped Sandpiper. Reconstructions were inferred using BSSVS with complete state-transition histories implemented in BEAST, with approximately 50 sequences used for this analysis. The black points indicate nodes with posterior probabilities > 0.95, and the light gray bars indicate 95% HPD intervals.

Since its first detection in Argentina, this H1 lineage has been reported only in Chile and Argentina, and now, for the first time, has been detected in Brazil. Bayesian temporal inference estimated the tMRCA of this South American lineage at 2005 (95% HPD [2002–2008]), with the South American clade recovered as monophyletic with maximal posterior support (posterior probability = 1.0) across all four demographic-and-clock model combinations (Figure 6). The lineage diverged from its North American relatives deep in the tree, with the root of the combined North–South American phylogeny dated to approximately 1974. Nucleotide identity with the first described Argentinian sequence, sampled from a red-winged tinamou in 2008, showed 93.4% nucleotide identity, consistent with prolonged independent divergence within South America.

Summarizing complete state-transition histories (Markov jumps) across the posterior, Chile-to-Argentina transitions were the most frequently reconstructed geographic jumps, converging with the BSSVS analysis, which recovered decisive support for movement from Chile to Argentina (BF 87–125 across model cells) and lower but consistent support for the reverse direction. This directionality was constant: the Chile to Argentina axis was recovered under all four model combinations and in all jackknife replicates (Figure 6, Supplementary File S3), and transitions with the highest Markov-jump counts were also those with the strongest Bayes-factor support, indicating agreement between the two measures. The generally higher support for Chile as a source, with Argentina also recovered as a supported origin, reflects the residual uncertainty regarding the precise geographic origin of dispersal within the southern cone rather than a single resolved route. Against this southern-cone background, the Brazilian isolate was recovered as a recent, terminal introduction most closely associated with Chilean viruses, though with the single Brazilian sequence, this placement should be read as the position of the isolate within the sampled diversity rather than as a resolved introduction route. Ancestral host-state reconstruction revealed that, despite an earliest inferred association with a Red-winged tinamou, the South American H1 lineage was predominantly maintained in waterfowl, particularly dabbling ducks, throughout its evolutionary history (Figure 6). Within this waterfowl reservoir, the asymmetric BSSVS framework identified the Yellow-billed teal as a central host hub, with the best-supported host-state transitions directed toward other dabbling ducks, most consistently the Yellow-billed pintail and Red shoveler. These teal-centred transitions were recovered across the four model combinations and the jackknife replicates, whereas transitions toward other hosts were less stable and should be interpreted with caution (Supplementary File S3). Furthermore, viral circulation in non-waterfowl hosts was extremely rare, limited to a Blackish oystercatcher (Chile, 2018), a Whimbrel (2016), and the H1N8 isolate in a White-rumped Sandpiper (Brazil, 2024).

The same geographic signal was recovered independently for the two locally acquired surface/accessory segments. Under the identical BSSVS framework, both NA and NS supported the Chile–Argentina axis with considerably higher BFs than HA (NA, Chile → Argentina BF = 360; NS, BF = 933 and 260 in the two supported directions; Supplementary File S4), and in both cases placed the Brazilian isolate as derived from southern-cone viruses (Figure 2 and Figure 3). Taken together, the three local segments reveal supported transitions among Southern Cone countries were consistently observed across independent genealogies, with the PNLP site in Brazil representing the sole Brazilian location and receiving well-supported transitions from these countries.

### 3.3. Transition States of the Shared Genomic Backbone

Across both skyride and coalescent models, temporal analyses estimated the tMRCA of the shared genomic backbone segments, represented by the PNLP viruses, and both H11N2 detected at Delaware, dated between 2020–2021 across the five internal gene segments (Table 2A). Discrete phylogeographic analyses recovered supported transitions among the Delaware, Minnesota, PNLP_H11N2, and PNLP_H1N8 states, with strength and direction varying across segments; these transitions describe connections between discrete lineage states rather than datable ancestor-to-descendant events. *PB2*, *PA*, and *MP* linked the Delaware state to both PNLP viruses, as well as the two PNLP viruses to each other, the strongest being Delaware → PNLP_H11N2 in *MP* (BF = 52.42) and PNLP_H11N2 → PNLP_H1N8 in *PB2* (BF = 19.04). NP differed, with stronger support for Delaware → PNLP_H1N8 (BF = 24.42) than for PNLP_H11N2 → PNLP_H1N8 (BF = 2.24), while PB1 showed the most heterogeneous pattern, involving Minnesota and both PNLP viruses, and recent shared ancestry between PNLP viruses (2021, 95% HPD 2020–2022). Rather than resolving a single directional pathway, these segment-specific differences indicate that the shared backbone is best interpreted as a set of closely related lineages descending from a common North American–associated gene pool (Table 2B).

### 3.4. Reassortment Inference with TreeSort

TreeSort analyses provided complementary methodological support, by statistically identifying branch-specific reassortment involving specific genomic segments. Across all analyses using each of the five shared internal gene segments as independent reference phylogenies, the algorithm consistently mapped the acquisition of the HA and NA segments to the evolutionary branch leading to the PNLP H1N8 isolate. Additionally, the NS segment was independently inferred as a reassorted acquisition in four of the five reference analyses. When PB1 was used as the reference, NP and NS were additionally recovered as reassorted, indicating a more complex acquisition history than initially resolved for this lineage. The high concordance among these independent reference topologies closely matched the phylogenetic incongruences initially observed across the segment-specific ML trees. The full annotated TreeSort output trees and the corresponding reassortment table are provided in Supplementary File S5 and deposited at GitHub repository.

## 4. Discussion

This study provides an evolutionary reconstruction of a reassortment event supported by multiple complementary analytical approaches, rather than evidence of broader population-level dynamics. Our analysis points to a coherent evolutionary scenario linking the previously described PNLP H11N2 virus and the H1N8 detected one year and three months later. Our findings should therefore be interpreted as a snapshot of an evolutionary event occurring within the South American avian influenza virus gene pool, rather than as a comprehensive model of viral circulation.

**Figure 7:**
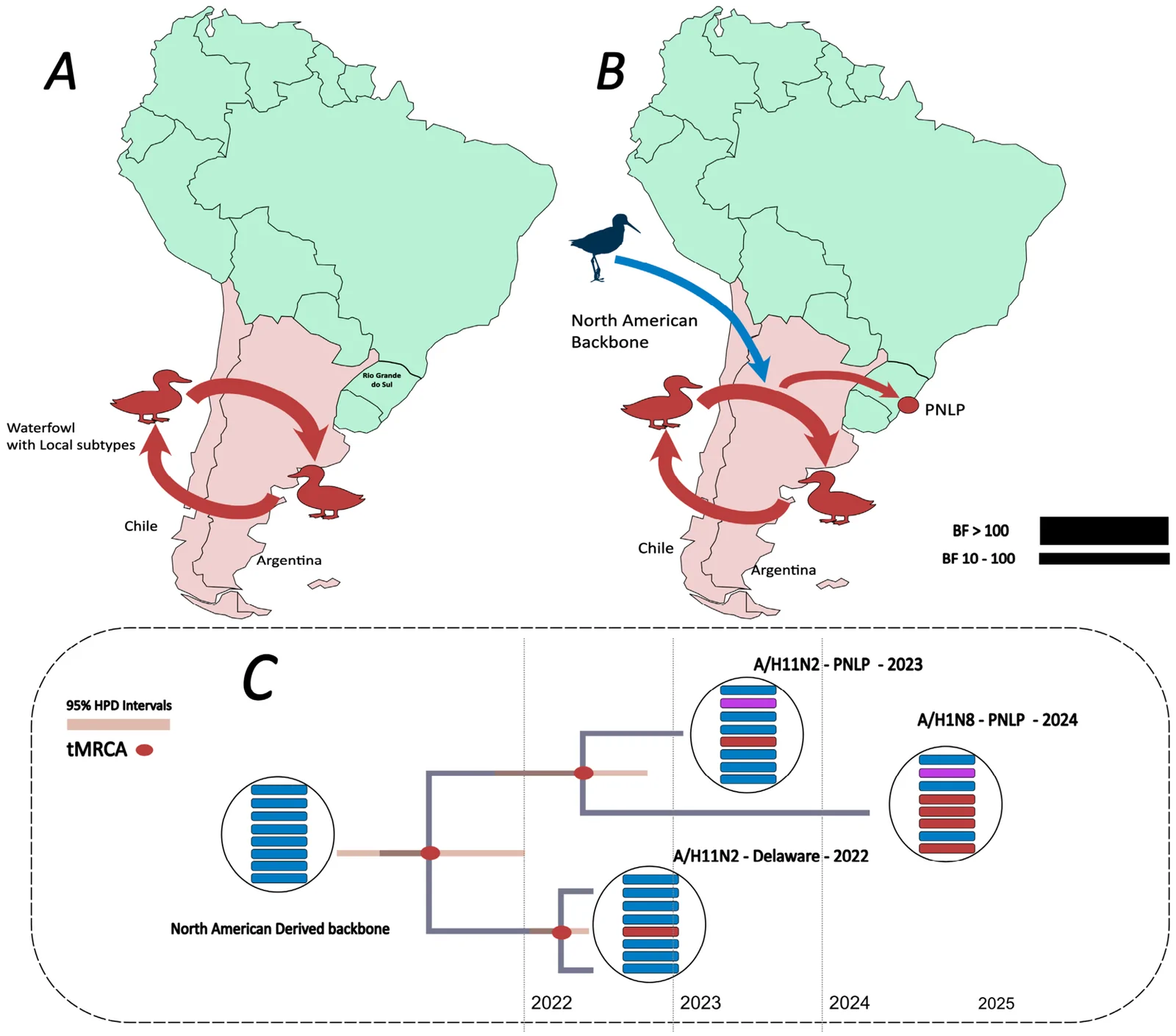
Proposed evolutionary model for the emergence of the reassortant H1N8 virus detected at Parque Nacional da Lagoa do Peixe (PNLP), southern Brazil. The figure presents an evolutionary hypothesis supported by the multi-segment analyses, not a resolved sequence of events. (**A**) Sustained local circulation of South American avian influenza virus lineages carrying local gene segments, with transitions annotated by the Bayes factors from the BSSVS analyses of the HA, NA, and NS segments. (**B**) Proposed reassortment between the North American-derived backbone, circulating in migratory shorebirds, and locally circulating South American viruses, whose precise location is not resolved by the data; arrows represent exchange among local subtypes and the entry of the Delaware-derived backbone into the region. (**C**) Time-scaled schematic of the shared *PB2* segment genealogy, tracing the genomic constellation from the North American-derived ancestral backbone through the Delaware A/H11N2 (2022) and the PNLP A/H11N2 (2023) to the reassortant A/H1N8 (2024). Internal nodes show their tMRCA (red points) and 95% HPD intervals (bars); the overlapping intervals indicate that a strict order of acquisition cannot be resolved. Red segments represent South American lineages, purple the PB1 lineage associated with HPAI H5N1 clade 2.3.4.4b (Am1.2), and blue North American-derived lineages. Created in Affinity Designer.

Across all analytical approaches a consistent pattern emerged: the *PB2*, *PB1*, *PA*, and *MP* segments trace to a North American-derived internal genomic backbone, dated to 2020–2021, that persisted locally and was independently detected in the PNLP H11N2 (2023) and H1N8 (2024) viruses, while the HA, NA, and NS segments were acquired from distinct, locally circulating South American lineages. The convergence of these independent lines of evidence is the central finding of this study. Rather than supporting a single direct reassortment event between the previously detected H11N2 and the H1N8 virus, the data indicate that the H1N8 genome arose on a shared internal genomic backbone onto which locally circulating South American gene segments were assembled through reassortment (Figure 7A–C).

The locally acquired genomic segments provide complementary insight into the long-term evolution of AIV in South America. The H1 lineage, in particular, showed evidence of prolonged circulation within the southern cone region, predominantly associated with waterfowl hosts. This geographic and host pattern is consistent with previous reports describing South American AIV lineages that are tied to specific locations and hosts and that evolved independently from their North American ancestors [7,9,21,47,48]. This southern-cone PNLP association was consistently recovered across all four demographic and clock model combinations and across the jackknife replicates and was independently reinforced by the NA and NS segments analyses, which recovered the same Chile–Argentina geographic signal from distinct genealogies, although surveillance gaps may still hide additional geographic patterns not represented in the current sampling.

Our findings reinforce this pattern of locally circulation, while also showing that such a lineage can, on occasion, emerge in new hosts and locations. Within the H1 lineage’s evolutionary history, non-waterfowl detections were rare, limited to a single Blackish Oystercatcher, Whimbrel and the White-rumped sandpiper itself, which are coastal birds rather than ducks. Such transitions into ecologically distinct, coastal hosts may reflect occasional ecological contact rather than an established route linking the dabbling-duck reservoir and shorebirds. While the BSSVS framework identified statistical support for a transmission link between these two detections, this pathway rests on the only two non-waterfowl records within the entire dataset and should accordingly be interpreted with caution, as an illustration of the rare, occasional spillover of this lineage beyond its waterfowl reservoir rather than as an established transmission route.

The NP segment showed a partially discordant history, with stronger support for a direct transition from the Delaware lineage to PNLP H1N8 than through PNLP H11N2 (Table 2). This suggests that the shared backbone may reflect multiple introductions or circulation of closely related descendants from the same North American ancestral gene pool, rather than a strictly linear H11N2-to-H1N8 evolutionary pathway. This segment also carries the deepest South American signal of the whole constellation. This lineage traces back to the earliest South American sequences (H7N3) sampled in Bolivia, 2001, and Chile, 2002 [48,49], placing the NP lineage within a deeply rooted regional clade (Figure 3). In this pool, the presence of a North American-derived backbone nested within this predominantly South American clade is a rare occurrence, of which the Delaware-associated PNLP lineage is one example, with sequences from Delaware Bay H11N2 detected in Semipalmated Sandpipers (*Calidris pusilla*, 2022) and a further Delaware H6N2 in 2024 (Figure 4). This NP cluster suggests multiple inter-hemispheric acquisitions within this lineage. Its detection in *Calidris* spp. across the Atlantic Flyway indicates this route may support bidirectional viral gene flow, rather than strictly one-way introductions. Although the exact ancestral sequence remains uncertain, a shared North American origin for the internal backbone is consistently supported.

A subtler form of segment-specific discordance was observed for PB1. This gene derived from the North American Am1.2 lineages that shares ancestry with the HPAI H5N1 clade 2.3.4.4b viruses [22], and it was the only shared segment for which Delaware was not recovered as a supported ancestral state; the signal involved Minnesota instead (Table 2). This conjuncture indicates a gene of a distinct North American lineage from the other four shared segments, and its persistence across both PNLP detections indicates that the shared backbone is not a geographically homogeneous unit, and it’s likely a product of reassortment events that occurred in the Northern continent. Additionally, its presence in both PNLP viruses points to a close relationship between them and suggests more complex reassortment history for this gene prior to its acquisition by the PNLP viruses, although the available data does not support any further inference regarding the specific reassortment events involved. Its persistence also suggests that segments sharing ancestry with the Am1.2 lineage associated with HPAI H5N1 B3.2 viruses remained present in LPAI viruses sampled from South American shorebirds, this observation concerns gene flow rather than phenotype and makes no inference about the pathogenicity of the H1N8 virus. However, we reinforce the value of characterizing internal gene segments in LPAI viruses instead of restricting genomic surveillance to the HA and NA subtypes.

Taken together, these results suggest a scenario in which the White-Rumped Sandpiper was the host where genetically distinct North and South American lineages were detected together. Through its long-distance migrations [50,51], this species was well positioned to “*fish*” for gene segments circulating in different regions and host communities from the Southern Cone wetland areas. The resulting H1N8 virus likely combined the locally circulating H1, N8, and NS lineages, maintained mainly in waterfowl, with the predominantly North American internal backbone already described in H11N2 viruses from Delaware and PNLP, itself linked to shorebird hosts. This contrast in host association between the acquired surface genes and the shared internal backbone illustrates the recurrent “mixing” of distinct AIV lineages that inter-hemispheric reassortment can produce.

However, the exact number of reassortments, precise ecological and geographical pathway underlying the emergence of this virus remain unresolved, and much of this interpretation is constrained by the limited availability of South American AIV genomes, which remain geographically and temporally sparse, resulting in substantial under sampling of local lineages. These gaps may also bias the geographic locations and hosts inferred as ancestral states, particularly for the H1 and N8 genes, for which available genomic sequences remain limited, resolving the real route of viral propagation, and whether shorebirds routinely participate in it, will require a substantially larger and more evenly distributed set of South American genomes than is currently available. Consequently, the evolutionary scenario proposed here should be regarded as the most parsimonious hypothesis supported by the currently available data, rather than a definitive reconstruction of viral history.

Nevertheless, this study illustrates how integrating complementary phylogenetic, phylodynamic, and reassortment analyses can substantially increase the evolutionary information extracted from viral detections, as already demonstrated in other studies of phylodynamics and phylogeography [32,33,34,52]. Despite differences in methodology, such integrative approaches represent an increasingly valuable strategy for understanding viral dispersal dynamics in data-sparse regions.

As genomic surveillance expands across South America, similar analytical frameworks could be applied to larger datasets to reconstruct viral dispersal with greater confidence and to evaluate reassortment processes at the population level. Recent genomic studies from South America have already demonstrated similar evolutionary approaches for reconstructing the initial dissemination of H5N1 clade 2.3.4.4b in the region [15,36], substantially improving our understanding of the dispersal of this virus across South America.

The recent detections of H1N8 at PNLP expand the list of AIV subtypes reported at this migratory site from Brazil (H11N2-1, H2N2-1, H6Nx-1, H6N1-8, H9N2-1, and H12N5-1) [21,22]. The identification of genetically related internal segments across different years supports the importance of PNLP for surveillance of AIV, especially for Nearctic migratory birds. Given the historically limited genomic data, such locations may harbor LPAI diversity that becomes apparent only through sustained monitoring. Notably, sampling was conducted during the H5N1 first incursion in the Americas [17], yet no HPAI was detected despite a high number of collected samples. On the other hand, it serves as evidence that not all coastal regions in Brazil were equally affected. Although this study is based on a limited number of isolates, many studies similarly report few recoveries despite extensive sampling efforts [53,54,55,56], and overall prevalence tends to be consistently low (often <1%).

Furthermore, although a large number of samples was collected, the majority consisted of environmental fecal material, a matrix that is naturally less sensitive for viral detection and recovery, as it contains inhibitors that reduce detection sensitivity and hinder successful isolation [57], and samples collected directly from individual birds, such as oropharyngeal and cloacal swabs, would likely have yielded a higher number of detections and viral recoveries.

## 5. Conclusions

The detection of the H1N8 virus expands the diversity of avian influenza virus subtypes documented at Parque Nacional da Lagoa do Peixe and reinforces the importance of this migratory hotspot as a long-term surveillance site in South America. Our analyses support that the PNLP H1N8 virus emerged through reassortment, combining locally circulating South American gene segments with the same predominantly North American-derived internal genomic backbone identified in the PNLP H11N2 lineage. By integrating multiple approaches, we reconstructed the most parsimonious evolutionary scenario for the origin of this reassortant despite the limited genomic representation of South American AIVs. These findings demonstrate the value of combining complementary evolutionary analyses to resolve reassortment histories and highlight the importance of sustained One Health genomic surveillance at major migratory interfaces for the early detection and characterization of emerging avian influenza viruses.

## Figures and Tables

**Table 1:** BlastN hits for each gene segment of the H1N8 virus isolated in this study, with corresponding geographic origin and nucleotide identity. Asterisks highlight the target isolates within the ML trees.

Gene Segment	Blast Hits	Geographic Origin	Identity (%)
*PB2*	A/white-rumped sandpiper/Brazil/PNLP_SM10/2023(H11N2)	Brazil (PNLP)	99%
*PB1*	A/white-rumped sandpiper/Brazil/PNLP_SM10/2023(H11N2) *	Brazil (PNLP)	99%
*PA*	A/white-rumped sandpiper/Brazil/PNLP_SM10/2023(H11N2)	Brazil (PNLP)	98%
*HA*	A/blackish oystercatcher/Chile/C38127/2018(H1N1) *	Chile	97%
*NP*	A/white-rumped sandpiper/Brazil/PNLP_SM10/2023(H11N2) *	Brazil (PNLP)	99%
*NA*	A/yellow-billed pintail/Argentina/CIP112-3626/2019(H3N8) *	Argentina	97%
*MP*	A/white-rumped sandpiper/Brazil/PNLP_SM10/2023(H11N2)	Brazil (PNLP)	99%
*NS*	A/environment/Chile/C63181/2023(H5N7) *	Chile	98%

**Table 2:** Bayesian estimates for the five shared internal gene segments of the PNLP H11N2 (2023) and H1N8 (2024) viruses. (**A**) Gene segments, its tMRCA and 95% HPD intervals of the shared backbone lineage; (**B**) BSSVS-supported transitions between discrete lineage states (Delaware, Minnesota, PNLP_H11N2, PNLP_H1N8), with their corresponding BF. Reported tMRCA values were derived from the Skyride analyses. Log and tree files are deposited at GitHub repository; complete BSSVS-supported transitions are provided in Supplementary File S4. Highest BF values are boxed.

A		B	
Gene	tMRCA of Shared Backbone [95% HPD Intervals)	Supported Ancestral Transition	Bayes Factor
***PB2***	**2021 [2020–2022]**	Delaware → PNLP_H11N2 Delaware → PNLP_H1N8 PNLP_H11N2 → PNLP_H1N8	7.47 3.87 19.04
***PB1***	**2021 [2020–2022]**	PNLP_H11N2 → PNLP_H1N8 Minnesota → PNLP_H11N2 PNLP_H1N8 → PNLP_H11N2	31.84 7.07 5.64
***PA***	**2021 [2020–2021]**	Delaware → PNLP_H11N2 Delaware → PNLP_H1N8 PNLP_H11N2 → PNLP_H1N8	9.20 4.31 6.17
***NP***	**2020 [2019–2021]**	Delaware → PNLP_H11N2 Delaware → PNLP_H1N8 PNLP_H11N2 → PNLP_H1N8	16.47 24.42 2.24
***MP***	**2020 [2018–2020]**	Delaware → PNLP_H11N2 Delaware → PNLP_H1N8 PNLP_H11N2 → PNLP_H1N8	52.42 8.01 15.04

## Data Availability

The full genome of the A/White-Rumped Sandpiper/Brazil/PNLP-222/2024 (H1N8) virus has been deposited in the GenBank database under the accession numbers: PX063867 to PX063874. Raw FASTQ sequencing reads and the complete BEAST X v.10.5 MCMC log and posterior tree files (.log–.trees) generated in this study are available on the Open Science Framework (OSF) platform (https://doi.org/10.17605/OSF.IO/HV68C (accessed on 1 September 2026). BEAST XML input files, maximum-likelihood and maximum clade credibility trees, TreeSort input/output files, and Sanger sequencing chromatograms are available in the GitHub repository https://github.com/gustavofn13/PNLP_H1N8_Pathogens (accessed on 1 September 2026).

## Supplementary Materials

The following supporting information can be downloaded at: https://www.mdpi.com/article/10.3390/pathogens15101039/s1. Figure S1. Field sampling and sample collection activities at the study site. (A) Shorebird captured during field sampling. (B) Researcher wearing personal protective equipment during bird handling. (C) Collection of a biological sample from a bird. (D) Bird observed at the sampling site. (E) Group of shorebirds present along the coastal area during field activities. Supplementary File S1: Surveillance dataset and positive sample characterization. (Table S1). Complete field log of the five sampling expeditions, detailing every sample collected (*n* = 1787), including species taxonomy, individual counts, capture date, sample type (swab/feces), geographic sampling coordinates, and molecular (RT-qPCR) test result. (Table S2) Individual metadata for the six AIV RT-qPCR-positive samples, including host species, Ct value, number of egg passages, and sequencing outcome. Supplementary File S2: Next-Generation Sequencing (NGS) metrics across all viral genome segments, including read counts, mean sequencing depth, and GenBank accession numbers; Supplementary File S3: HA BEAST reports, summarizes the estimated state transition counts and mean rates for the hemagglutinin gene, from South and North American countries included in the dataset across different clock models and tree priors, mapping viral dispersal events across distinct hosts and geographic locations; Jackknife reports are also included in this analysis. Supplementary File S4: BSSVS, reports Bayesian Stochastic Search Variable Selection results across viral segments, presenting posterior probabilities and Bayes Factors (BF) supporting inferred transmission routes. Supplementary File S5: TreeSort analyzed files, containing alignment and annotated tree files that were used for analysis. A summarizing table containing the results is also included.
